# Alveolar epithelial cells in bacterial sepsis-associated acute lung injury: mechanisms and therapeutic strategies

**DOI:** 10.3389/fimmu.2025.1605797

**Published:** 2025-08-06

**Authors:** Guiyang Jia, Erqin Song, Zhiyou Zheng, Mingjiang Qian, Guoyue Liu

**Affiliations:** ^1^ Department of Critical Care Medicine, The Second Affiliated Hospital of Zunyi Medical University, Zunyi, China; ^2^ Emergency Department, The Second Affiliated Hospital of Zunyi Medical University, Zunyi, China

**Keywords:** alveolar epithelial cells, bacterial sepsis-associated acute lung injury, pathophysiology, mechanisms, treatment progress

## Abstract

Bacterial Sepsis-Associated acute lung injury (ALI) and its progression to acute respiratory distress syndrome (ARDS) are clinically prevalent critical conditions with high morbidity and mortality. As a vital component of lung tissue, alveolar epithelial cells (AECs) play a crucial role in maintaining pulmonary homeostasis and are deeply involved in the pathophysiological processes of bacterial Sepsis-Associated ALI. This review systematically summarizes the pathophysiological changes in AECs during bacterial sepsis, focusing on oxidative stress, programmed cell death, and disruption of the epithelial barrier. It further explores the inflammatory responses triggered by both Gram-positive and Gram-negative bacteria, as well as the interactions between AECs and immune cells, shedding light on how these processes contribute to the inflammatory response during bacterial sepsis. It elaborates on the regulatory mechanisms of key molecular pathways, including Nuclear factor kappa-B (NF-κB), Nuclear Factor Erythroid 2-related Factor 2 (NRF2), nucleotide-binding oligomerization domain-like receptor family pyrin domain-containing 3 (NLRP3), and Toll-like receptor (TLR), in AEC dysfunction and inflammatory responses. Furthermore, therapeutic strategies for AEC injury are comprehensively analyzed from multiple perspectives, such as AEC repair and regeneration, modulation of inflammatory responses, restoration of barrier function, and exosome-based therapies. Although these approaches show promising results in preclinical studies, their clinical translation faces significant challenges. This review underscores the need for further research into the complex mechanisms of AEC injury in bacterial sepsis and advocates for the development of more targeted interventions to improve patient outcomes.

## 1 Introduction

The lungs are essential organs in the human body, and injury to lung tissue, along with subsequent respiratory failure, constitutes a significant clinical concern. Severe cases of lung injury may progress to acute respiratory distress syndrome (ARDS), exacerbating the underlying condition, complicating treatment efforts, and increasing mortality rates (1). Previous studies have indicated that ARDS develops as a progression of acute lung injury (ALI). Research has identified several factors that contribute to ALI, including infectious agents such as pulmonary (2) and non-pulmonary sepsis (3), as well as non-infectious causes like trauma, electronic cigarette exposure (4), mechanical ventilation (5), pancreatitis, and blood transfusion. These factors induce ALI through both localized inflammatory responses and systemic inflammation, impairing respiratory function. Among these, sepsis remains the most common and severe cause of ALI (6). Sepsis is a critical condition triggered by immune dysfunction induced by infections leading to widespread systemic inflammation and organ failure, with the lungs frequently affected (7). The inflammation associated with sepsis can directly or indirectly damage endothelial cells (8) and alveolar epithelial cells (AECs) (9) via mechanisms such as oxidative stress, inflammatory responses, and disturbances in microcirculation. These processes result in vascular leakage, pulmonary edema, reduced alveolar surfactant, and alveolar collapse, which ultimately leads to respiratory failure (10, 11). In addition, AECs interact with alveolar macrophages to secrete various substances that trigger immune responses to defend against pathogens and clear particulate matter (12). Research on ALI has highlighted the involvement of AECs in the inflammatory process (13, 14), with studies showing that preventing AEC death can effectively reduce ALI and improve patient prognosis (15).

This review investigates the pathophysiological role of AECs in bacterial Sepsis-Associated ALI. It provides a comprehensive overview of the alterations in AECs that occur during the onset of bacterial Sepsis-Associated ALI, highlighting the underlying molecular mechanisms. The review also explores various therapeutic approaches and potential targets for modulating AEC function and activity to mitigate the impact of sepsis-associated ALI. These insights offer valuable perspectives for advancing research and mitigating the progression of sepsis-associated ALI to acute respiratory distress syndrome (ARDS).

## 2 Physiological function of AECs

**Figure 1:**
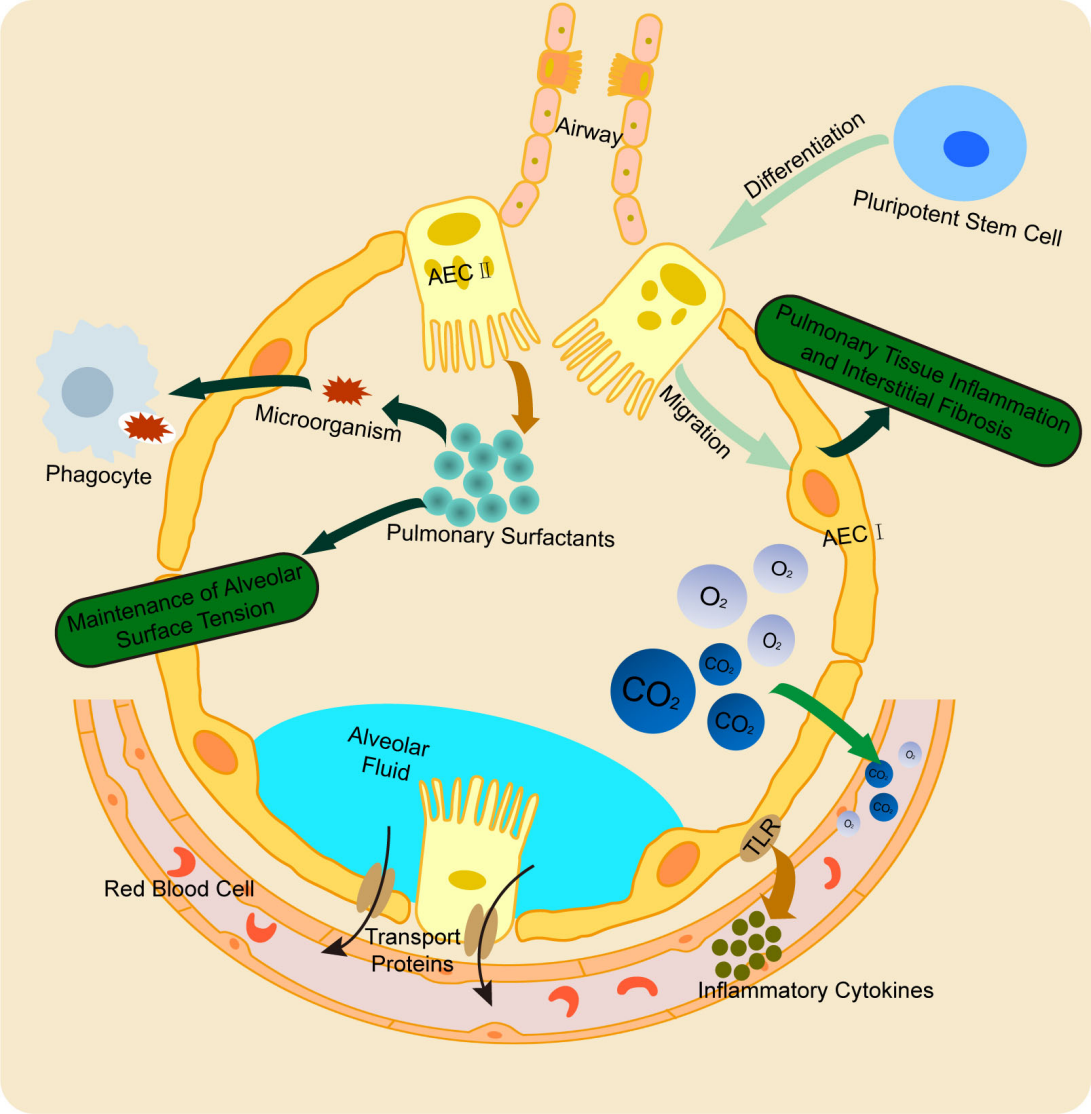
Physiological function of AECs. AEC I and AEC II cooperatively maintain alveolar structure and regulate fluid clearance. AEC I primarily mediate gas exchange and participate in inflammatory responses, while AEC II synthesize pulmonary surfactant to stabilize alveoli. Notably, AEC II possess progenitor capacity, enabling their differentiation into AEC I to repair damaged epithelium. This synergistic interaction ensures proper alveolar function and integrity.

The alveoli are the terminal respiratory units of the lung, playing a crucial role in maintaining normal respiratory function and facilitating gas exchange. AECs, which are essential components of the alveolar structure, are classified into type I alveolar epithelial cells (AEC I) and type II alveolar epithelial cells (AEC II) pneumocytes (16). AEC I are derived from the differentiation of multipotent stem cells into AEC II, followed by transdifferentiation. These cells form the majority of the alveolar epithelium. AEC I, with their unique flattened morphology, enable efficient gas diffusion into the capillaries, maintain ion balance at the alveolar air-liquid interface, and support optimal gas exchange (17). In addition to their structural role, AEC I are involved in inflammatory responses and apoptosis. A deficiency in AEC I function can lead to inflammation, defective tissue repair, impaired remodeling, and interstitial fibrosis, contributing to chronic pulmonary diseases such as chronic obstructive pulmonary disease (COPD) and pulmonary fibrosis (18). AEC I express Toll-like receptors (TLRs) and secrete pro-inflammatory cytokines in response to injury, modulating immune responses. AEC II, on the other hand, are responsible for synthesizing pulmonary surfactant, a mixture of proteins and phospholipids that binds microbial components (such as bacterial, fungal, and viral lipids/proteins) to facilitate phagocytic clearance and enhance lung immunity (19). Surfactant produced by AEC II reduces alveolar surface tension, stabilizes the epithelial barrier, and prevents alveolar collapse, thus preserving lung architecture (20). Notably, AEC II possess progenitor cell properties. In response to AEC I injury or death, they proliferate and differentiate into AEC I to repair the alveolar epithelium. This regenerative function underscores the critical role of AEC II in maintaining epithelial integrity (21). Together, the AEC I-AEC II epithelium forms a tightly regulated barrier that allows for efficient gas exchange while preventing the passage of excess water, electrolytes, and hydrophilic solutes. The functional synergy between AEC I and AEC II, coupled with the integrity of their junctional complexes, ensures the coordination of gas exchange and alveolar fluid clearance (22) (**Figure 1**).

## 3 Pathophysiology of AECs in bacterial sepsis-associated ALI

**Figure 2:**
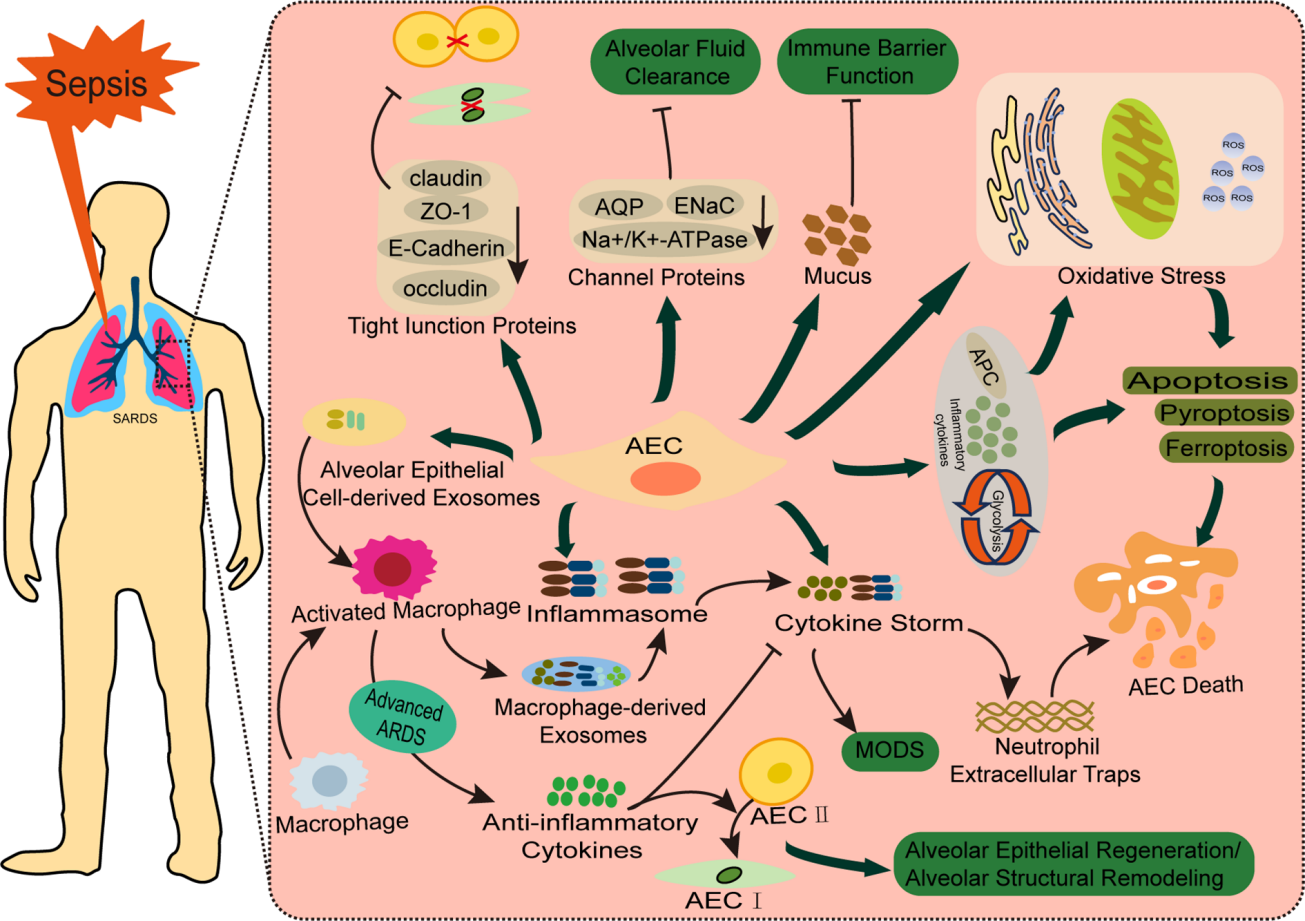
Pathophysiology of AECs in bacterial sepsis-associated ALI. Bacterial sepsis triggers AEC injury through two major pathways: (1) Cellular dysfunction and structural damage, involving oxidative stress, multiple cell death pathways (apoptosis, pyroptosis, ferroptosis, and cuproptosis), and disruption of tight junctions (ZO-1, claudins) and ion channels (ENaC, AQPs) that impair alveolar barrier function and fluid clearance; (2) Inflammatory amplification through cytokine release and crosstalk with immune cells (macrophages and neutrophils). These synergistic mechanisms collectively contribute to the development and progression of bacteria Sepsis-Associated ALI.

AEC injury plays a critical role in the pathogenesis and progression of bacterial sepsis-Associated ALI. In Sepsis-Associated ALI, the structural integrity of the alveolar walls is compromised. AEC I undergoing cytoplasmic swelling, blebbing, and rupture. Meanwhile, AEC II exhibits cytoplasmic shrinkage, disorganized structures with vacuolar degeneration, depletion and necrosis of lamellar bodies, and lysosomal proliferation. Additionally, AEC II show a reduction or complete loss of microvilli, mitochondrial cristae destruction, endoplasmic reticulum dilation, altered nuclear morphology, and prominent perinuclear spaces. Apoptotic nucleoli, secretory granules, and apoptotic bodies are also observed (23, 24). Infection by bacterial pathogens, immune complexes, extracellular vesicles (EVs) released by immune cells, and bacterial endotoxins contribute to AEC injury and dysfunction. These factors induce oxidative stress, promote various forms of programmed cell death, including apoptosis, necroptosis, and pyroptosis, impair cellular viability and proliferation (25–27), and disrupt intercellular junctions and the epithelial barrier function (28). These mechanisms also facilitate epithelial-mesenchymal transition (EMT) (29), exacerbating lung injury. Moreover, interactions between AECs and immune cells during immune-inflammatory responses can drive systemic inflammation (30), further increasing the risk of ALI or directly damaging lung tissue (31, 32) (**Figure 2**).

### 3.1 AEC injury and dysfunction in sepsis

#### 3.1.1 Oxidative stress and cell death

Oxidative stress and mitochondrial dysfunction are important mechanisms of cellular injury, contributing to the pathogenesis of various pulmonary diseases, including COPD, idiopathic pulmonary fibrosis (IPF), and ALI (33). In bacterial sepsis-induced AEC injury, bacterial components and endotoxins trigger endoplasmic reticulum stress and dysregulated autophagy in AECs (34). These pathological changes lead to the excessive production of reactive oxygen species (ROS), reactive nitrogen species (RNS), and lipid peroxidation products, in conjunction with a weakened antioxidant defense system (35). As a result, mitochondrial membrane potential depolarization and functional impairment occur, disrupting cellular energy metabolism and promoting AEC apoptosis (36). Therapeutic interventions targeting these pathways—such as antioxidant therapy, modulation of mitophagy (37, 38), and the regulation of oxidative stress and mitochondrial function (39, 40)—can alleviate sepsis-induced AEC injury by promoting alveolar epithelial cell proliferation, inhibiting apoptosis and ferroptosis, suppressing inflammatory responses, and reducing oxidative stress, thereby mitigating bacterial Sepsis-Associated ALI.

Cell death is generally categorized into programmed cell death (including apoptosis, necroptosis, and pyroptosis) and metabolism-related cell death (such as ferroptosis and cuproptosis), that arise due to metabolic dysregulation. These mechanisms are essential for the removal of damaged or senescent cells, thereby ensuring normal growth and development. However, dysregulated or excessive activation of these pathways can contribute to the pathogenesis of various diseases (41).

In the context of bacterial Sepsis-Associated ALI, research has shown that endotoxins, certain bacterial pathogens, and immune complexes can promote apoptosis in AECs while inhibiting AEC proliferation. These effects are mediated through multiple mechanisms, including modulation of glycolysis and inflammatory responses (42), inhibition of activated protein C (43), and disruption of heat shock proteins (44). These factors all enhance apoptosis-inducing factors and worsen Sepsis-Associated ALI progression (45, 46). Notably, pharmacological inhibition of AEC apoptosis has been found to alleviate ALI in experimental models, improving survival outcomes (47, 48). Endotoxins can also activate canonical pyroptosis-related molecules in AECs, such as nucleotide-binding oligomerization domain-like receptor family pyrin domain-containing 3 (NLRP3) and gasdermin D (GSDMD), promoting inflammatory responses and contributing to cellular injury (49). Inhibition of the activation of these pyroptosis-related molecules can alleviate AEC damage, thereby mitigating bacterial Sepsis-Associated ALI (50, 51).

Ferroptosis has emerged as a key form of metabolic cell death in AECs during bacterial Sepsis-Associated ALI. Experimental findings demonstrate that endotoxins induce ferroptosis in AECs by disrupting the expression of ferroptosis-related proteins, such as Glutathione Peroxidase 4 (GPX4), Solute Carrier Family 7 Member 11 (SLC7A11), and Ferritin Heavy Chain 1 (FTH1). This leads to characteristic mitochondrial alterations in AECs, including shrinkage, reduced size, and a loss of cristae (52). Furthermore, in the Cecal ligation and puncture (CLP) mouse model, the release of neutrophil extracellular traps (NETs) can induce ferroptosis in AECs by regulating the stability of Hypoxia-Inducible Factor-1 Alpha (HIF-1α), thereby exacerbating ALI (53). Pharmacological modulation of the ferroptosis pathway showed therapeutic potential, enhancing cellular viability and significantly reducing pulmonary inflammation and ALI in septic animal models (54, 55). Cuproptosis represents a recently identified form of metabolic cell death. Current bioinformatic studies reveal its involvement in bacterial sepsis pathogenesis, particularly demonstrating complex relationships with bacterial Sepsis-Associated ALI (56, 57). However, the precise mechanisms through which cuproptosis contributes to bacterial Sepsis-Associated ALI, including its specific interactions with AECs, require further elucidation.

In summary, programmed and metabolically regulated cell death pathways in AECs constitute significant pathophysiological alterations contributing to the development of bacterial Sepsis-Associated ALI. While current research has yielded insights into apoptosis and ferroptosis, a definitive consensus on their precise roles and mechanisms remains elusive, and the translation into effective clinical therapeutics necessitates further investigation. Furthermore, the contributions of emerging cell death modalities—such as necroptosis, pyroptosis, and cuproptosis, to the pathogenesis of bacterial Sepsis-Associated ALI warrant deeper exploration.

#### 3.1.2 Breakdown of epithelial barriers and tight junctions

AECs are essential structural components of the alveoli, crucial for maintaining normal pulmonary function through their intercellular junctions and barrier properties. Recent studies have shown that AECs from septic patients secrete mucus containing carbohydrates such as N-acetyl-galactosamine, galactose, and N-acetyl-neuraminic acid. These alterations in carbohydrate composition disrupt lectin-binding patterns, impairing the mucosal immune barrier and resulting in delayed or defective protective responses (58). Pathogenic factors, including bacteria and endotoxins, significantly reduce the expression of tight junction proteins (such as ZO-1 (Zonula Occludens-1), occludin, claudin-1, claudin-3, and claudin-18) and adherens junction proteins (E-cadherin) in AECs. This disruption of intercellular connections compromises alveolar barrier integrity, increases permeability, and exacerbates pulmonary exudation and pathological damage in bacterial sepsis (28, 59, 60). *In vitro* co-culture experiments have shown that AEC-derived soluble protective factors can attenuate lipopolysaccharide (LPS)-induced migration of human pulmonary microvascular endothelial cells and neutrophils, while reducing endothelial permeability and albumin leakage (61). Moreover, endotoxins downregulate the expression and accelerate the degradation of surfactant proteins (62) and key ion channels/water transporters - including the Epithelial Sodium Channel (ENaC), Sodium-Potassium ATPase (Na+/K+-ATPase), and Aquaporin-1/5 (AQP1/5) - in AECs. These molecular changes impair alveolar fluid clearance, contribute to pulmonary edema, and exacerbate exudation, ultimately facilitating the development of bacterial Sepsis-Associated ALI (63–65).

In conclusion, oxidative stress, programmed cell death, and dysfunction of intercellular junctions and barrier properties in AECs play central roles in the pathogenesis of bacterial Sepsis-Associated ALI. Therapeutic strategies aimed at restoring AEC viability and barrier function represent promising approaches for treating bacterial Sepsis-Associated ALI. However, further research is needed to develop specific pharmacological modulators and clarify their mechanisms of action.

### 3.2 Bidirectional interaction between AECs and inflammation in bacterial sepsis-associated ALI

While the systemic inflammatory response induced by bacterial sepsis is a well-recognized contributor to AEC injury, emerging evidence suggests that AECs themselves can actively participate in the inflammatory cascade. Under certain pathological conditions, AECs are capable of producing pro-inflammatory cytokines and chemokines, thereby amplifying systemic inflammation and contributing to multi-organ damage in conditions such as sepsis, ALI, and ischemia-reperfusion injury (66). Furthermore, AECs engage in crosstalk with immune cells, modulating both local pulmonary inflammation and broader immune responses. These interactions can disrupt alveolar structure and impair respiratory function, further exacerbating disease progression (67).

#### 3.2.1 AEC and inflammation

Inflammation is a key pathogenic mechanisms in bacterial Sepsis-Associated ALI. Research has found significant differences in the mechanisms of neutrophil infiltration in the alveoli caused by different pathogens (Gram-positive or Gram-negative bacteria). For instance, Gram-negative bacteria can mediate infiltration through CD18 or β2 integrins, whereas Gram-positive bacteria use CD29 or β1 integrins. In ALI associated with pneumonia, inflammation is often observed to resolve, whereas Sepsis-Associated ALI lacks this resolution, suggesting differences in the lung injury caused by sepsis depending on the infection site and pathogen (68).

In CLP or LPS-induced bacterial sepsis models, it was found that suppression of Sirtuin 1 (SIRT1) expression in AECs (69) or direct activation of the inflammasome (14) amplifies inflammatory signaling, triggering a cascade of cytokines and chemokines that further disrupt immune regulation and promote multi-organ dysfunction (30, 70). In contrast, therapeutic strategies aimed at reducing inflammatory cytokine release from AECs have been shown to attenuate pulmonary inflammation and alleviate bacterial Sepsis-Associated ALI pathology (71, 72).

Staphylococcus aureus represent the predominant Gram-positive bacterial pathogens implicated in bacterial Sepsis-Associated ALI. Current research has demonstrated that peptidoglycan (PGN) from the Staphylococcus aureus cell wall can activate critical transcription factors including AP-1, Nuclear factor kappa-B (NF-κB), and Nuclear Factor for Interleukin-6 (NF-IL6) (73). Additionally, AECs serve as highly responsive targets for Staphylococcus aureusα-toxin, which hydrolyzes phosphatidylinositol (PtdIns) within these cells, triggering the release of nitric oxide, prostaglandins (PGE2, PGI2), and thromboxane A2 (TxA2), thereby exacerbating the pulmonary inflammatory response (74).

In summary, irrespective of the causative Gram-positive or Gram-negative bacterium, AECs in bacterial sepsis function dually as targets of inflammatory assault and potent sources of inflammatory cytokines. Consequently, targeting the regulation of AEC-associated inflammatory responses presents a potential therapeutic strategy for mitigating bacterial Sepsis-Associated ALI/ARDS and the systemic inflammatory response syndrome (SIRS) in sepsis.

#### 3.2.2 Interaction of AEC with immune cells

Macrophages are key regulators of innate immunity, and play a central role in the dysregulated inflammatory responses characteristic of bacterial sepsis (75). Under physiological conditions, AECs and macrophages sustain pulmonary homeostasis via ligand-receptor-mediated interactions. However, during the early stages of ALI, endotoxins can regulate mitochondrial fusion protein Optic Atrophy 1 (OPA1) acetylation through Sirtuin 3 (SIRT3), thereby modulating mitochondrial dynamics and promoting macrophage activation. This leads to a shift from the anti-inflammatory M2 phenotype to the pro-inflammatory M1 phenotype, which results in the secretion of pro-inflammatory cytokines. Macrophages also activate inflammasomes in AECs through exosome release, impairing AEC barrier function and inducing pyroptosis (76, 77). In both cell and animal studies, endotoxins have also been shown to promote the release of exosomal mediators such as Tenascin-C (TNC) and miR-92a-3p from AECs. These mediators activate the NF-κB inflammatory signaling pathway in macrophages, enhancing the release of inflammatory cytokines and inducing pulmonary tissue inflammation (78, 79). Recent studies have found that several targets, including NF-κB, Nuclear Factor Erythroid 2-related Factor 2 (NRF2), Signal Transducer and Activator of Transcription 1 (STAT1), Interferon Regulatory Factor 1 (IRF1), and Peroxisome Proliferator-Activated Receptor Gamma (PPARγ), are associated with both macrophage polarization and AEC injury in the context of bacterial Sepsis-Associated ALI. However, the specific roles and regulatory mechanisms of these targets in the interaction between AECs and macrophage polarization remain to be further explored (80, 81). In the late stage of ALI, anti-inflammatory cytokines secreted by selectively activated macrophages suppress the inflammatory response, promote AEC II proliferation, and facilitate differentiation into AEC I, contributing to alveolar epithelial regeneration and structural remodeling (82). These findings highlight a dynamic and bidirectional interaction between macrophages and AECs in bacterial Sepsis-Associated ALI (83). On one hand, macrophages release pro-inflammatory cytokines that damage AECs, while AECs, in turn, release exosomes that act back on macrophages, promoting the release of inflammatory cytokines. On the other hand, macrophages also promote alveolar epithelial regeneration and structural remodeling, protecting alveolar structure and function. Additionally, studies have found that endotoxins can enhance the expression of intercellular adhesion molecule-1 (ICAM-1) in lung tissue and AECs, increasing neutrophil adhesion to AECs, promoting neutrophil infiltration and exacerbating the inflammatory response in injured alveoli (84). AECs are closely interact with macrophages, neutrophils, and other immune cells. However, regulating these interactions to alleviate bacterial Sepsis-Associated ALI requires further in-depth research.

In conclusion, AECs serve as both targets of bacterial sepsis-induced systemic inflammatory damage and active participants in the pathological process through the secretion of inflammatory mediators, exosomes, and their dynamic interactions with immune cells. Modulating AEC-derived inflammatory factor release, exosome communication, and AEC-immune cell interactions presents a promising therapeutic strategy to mitigate systemic inflammation in bacterial sepsis and alleviate ALI. This dual approach, targeting both AEC dysfunction and their immunomodulatory roles, may offer novel therapeutic avenues for managing the complex pathophysiology of bacterial Sepsis-Associated ALI. However, current research is still at the basic research stage. Further clinical studies are needed to analyze the correlation between exosomal proteins such as TNC, miR-92a-3p, inflammatory cells, and inflammatory cytokines in bronchoalveolar lavage fluid and peripheral blood of patients with bacterial Sepsis-Associated ALI. These findings will help validate the results of basic research and lay the groundwork for future clinical applications.

## 4 Key mechanisms

### 4.1 NF-κB and phosphorylation

NF-κB plays a pivotal role in various physiological and pathological processes, including immune and inflammatory responses, cell survival, proliferation, and metabolism. It is critical in cellular responses to external stimuli, such as cytokines, stress, and antigens (85). In the context of bacterial Sepsis-Associated ALI, endotoxins induce AEC apoptosis and inflammatory responses through the regulation of the NF-κB/p65 signaling pathway (86), thereby inhibiting AEC apoptosis mediated by cytochrome C and caspase-3. Targeting NF-κB protein levels (87, 88) or inhibiting its activity (89) can reduce AEC inflammatory injury and alleviate bacterial Sepsis-Associated ALI. Further studies have shown that during septic inflammatory storms, NF-κB activation enhances the release of inflammatory mediators - including TNF-α, IL-1β, and Monocyte Chemoattractant Protein-1 (MCP-1) - while simultaneously promoting NET formation through NLRP3 inflammasome activation, thereby exacerbating pulmonary inflammation (90). Notably, NETs also facilitate m6A methylation modification, leading to the degradation of GPX4 mRNA via the Toll-like Receptor 9 (TLR9)/Myeloid Differentiation Primary Response 88 (MyD88)/NF-κB/methyltransferase-like 3 (METTL3) signaling pathway, thus reducing GPX4 protein expression and promoting AEC ferroptosis (72, 91). Reticulocalbin 3 (Rcn3) regulates the NF-κB/NLRP3/inflammasome axis, thereby protecting AECs and alleviating bacterial Sepsis-Associated ALI (92). Endotoxin-stimulated AEC exosomes secrete TNC, which binds to Toll-like Receptor 4 (TLR4) receptors and activates the P38/extracellular signal-regulated kinase (ERK)/NF-κB pathway, driving M1 macrophage polarization and inducing macrophage pyroptosis, thereby further intensifying inflammatory responses in bacterial Sepsis-Associated ALI (78). Additionally, PGN from Staphylococcus aureus cell walls can induce IL-8 expression through CD14 enhancement, ultimately activating NF-κB in AECs and promoting the development of bacterial Sepsis-Associated ALI (73). Cell and animal experiments have also confirmed that miR-92a-3p in AEC exosomes targets Phosphatase and Tensin Homolog (PTEN), activating the NF-κB/p65 signaling pathway in macrophages via the Phosphoinositide 3-Kinase (PI3K)/Protein Kinase B (Akt) pathway, thereby promoting the release of inflammatory cytokines and inducing pulmonary inflammation (79). Anemonin can inactivate NF-κB, thereby inhibiting lipopolysaccharide-induced AEC inflammation and oxidative stress, and alleviating bacterial Sepsis-Associated ALI (93). These findings underscore the central role of NF-κB in orchestrating complex inflammatory networks through various cellular and molecular mechanisms in the pathogenesis of bacterial Sepsis-Associated ALI.

Phosphorylation is the most prevalent post-translational modification, playing a critical role in regulating protein function and cellular functions. Dysregulated phosphorylation contributes to various diseases by affecting cell signaling, transmembrane protein synthesis, and key biological pathways, including apoptosis and autophagy (94). Phosphorylation is closely linked to the regulation of NF-κB in bacterial Sepsis-Associated ALI. Studies have shown that endotoxins activate ERK1/2 and p38/MAPK phosphorylation, leading to the translocation of cytoplasmic NF-κB to the nucleus and the activation of NF-κB activity. This may increase the expression of immune response-related TLR2 and modulate the expression of surfactant protein A (SP-A), which exacerbates bacterial Sepsis-Associated ALI (95). In contrast, activation of PI3K-Akt phosphorylation negatively regulates NF-κB phosphorylation, thereby inhibiting AEC apoptosis, alleviating bacterial Sepsis-Associated ALI, and reducing mortality in experimental models (96, 97). Additionally, modulation of P65 phosphorylation, a key factor in the NF-κB signaling pathway, has been shown to mitigate bacterial sepsis-induced AEC injury and reduce pulmonary inflammation in septic mice (98). Moreover, endotoxins promote c-Jun N-terminal Kinase (JNK) signaling pathway phosphorylation in a time-dependent manner, leading to AEC apoptosis (99).

In summary, modulation of the NF-κB signaling pathway and its phosphorylation is one of the key targets for regulating AEC injury and repair in bacterial Sepsis-Associated ALI. Recent treatments targeting the NF-κB signaling pathway to alleviate ALI have also proven effective in the treatment of Coronavirus Disease 2019 (COVID-19) (100). However, due to the significant Heterogeneity of ALI caused by different factors, the effectiveness of these drugs in ALI caused by various factors still requires further research. Additionally, activation of the canonical NF-κB/p65 pathway in AECs regulates the expression of coagulation and fibrinolysis-related proteins, such as tissue factor (TF) and plasminogen activator inhibitor-1 (PAI-1) (101). However, the precise molecular mechanisms underlying these effects require further investigation.

### 4.2 NRF2

NRF2 is the master regulator of the antioxidant response element (ARE) pathway. By binding to AREs in target gene promoters, NRF2 coordinates the expression of various cytoprotective enzymes and proteins, playing a critical role in redox homeostasis, oxidative stress defense, and cellular stress responses (102). In bacterial Sepsis-Associated ALI, endotoxins have been shown to suppress the NRF2/heme oxygenase-1 (HO-1) signaling pathway, exacerbating AEC inflammation, oxidative stress, and ferroptosis. Conversely, several agents, including AU-rich element ARE-binding factor 1 (AUF1) (103), mesenchymal stem cell (MSC)-derived exosomes (88), ferulic acid (104), and sufentanil (105), Ciprofol (106), and Anemonin (93) activate NRF2, thereby inhibiting AEC inflammation, oxidative stress, and cellular ferroptosis and apoptosis, ultimately alleviating bacterial Sepsis-Associated ALI progression. NRF2 also promotes the production of IL-17D in AECs, exerting a protective role in bacterial Sepsis-Associated ALI (107). Furthermore, NRF2 is involved in METTL4-mediated m6A methylation modification, thereby regulating mitochondrial homeostasis and cellular ferroptosis in bacterial Sepsis-Associated AECs (9). MSC-derived exosomes activate the NRF2 pathway, regulating processes like mitochondrial biogenesis and fission, maintaining mitochondrial function homeostasis, reducing LPS-induced AEC apoptosis, and alleviating bacterial Sepsis-Associated ALI in mice (108). Lysophosphatidylcholine (LPC) 14:0, by activating the NRF2/HO-1 signaling pathway, can also inhibit LPS-induced degradation of tight junction proteins, protecting AEC barrier function (109). These findings suggest that NRF2 plays a key role in the regulation of inflammation, oxidative stress, mitochondrial homeostasis, and barrier function maintenance in sepsis-related AEC injury. Targeting NRF2 could impact multiple aspects of bacterial sepsis-associated AEC damage, making it a potential therapeutic target for bacterial Sepsis-Associated ALI.

### 4.3 NLRP3 inflammasome

NLRP3 inflammasome is a multiprotein complex that plays a pivotal role in regulating inflammatory responses and processes such as pyroptosis through its signaling, regulatory, and effector functions (49, 110). In bacterial sepsis, endotoxins activate the NLRP3 inflammasome in both macrophages (76, 111) and AECs (14, 112), promoting AEC apoptosis, pyroptosis, and the release of inflammatory cytokines. This activation amplify inflammatory cell infiltration and exacerbate lung injury. This activation triggers programmed cell death and the release of pro-inflammatory cytokines, which in turn amplify inflammatory cell infiltration and exacerbate lung injury. Therapeutic strategies aimed at inhibiting NLRP3 activation, such as promoting NLRP3 degradation (51) or using anti-inflammatory compounds like Phillyrin (a natural lignan derived from *Forsythia*) (50) and Calycosin (an extract from *Astragalus membranaceus*) (72), have shown efficacy in reducing inflammatory cytokine production and the release of NETs. These interventions help alleviate AEC pyroptosis, apoptosis, and inflammatory responses, thereby mitigating the progression of bacterial Sepsis-Associated ALI.

In summary, in addition to regulating inflammatory responses, NLRP3 also plays a key role in apoptosis, pyroptosis, and other forms of programmed cell death in bacterial Sepsis-Associated AEC injury. Its mechanism may be related to the formation of the PANoptosome, which drives cell pyroptosis, apoptosis, PANoptosis, and necroptosis (113, 114). However, the specific molecular mechanisms and how NLRP3 regulates these processes in bacterial Sepsis-Associated ALI still require further investigation.

### 4.4 HMGB

High-mobility group (HMG) proteins are a family of evolutionarily conserved, non-histone nuclear proteins characterized by their high electrophoretic mobility. Among them, high-mobility group box (HMGB) proteins are the most abundant, comprising four isoforms: HMGB1, HMGB2, HMGB3, and HMGB4 (115). In bacterial Sepsis-Associated ALI, LPS promotes AEC inflammation and apoptosis via the HMGB1/receptor for advanced glycation end products (RAGE) pathway (116). Pharmacological agents with anti-inflammatory properties, such as calycosin (72) and propofol (89), have been shown to mitigate sepsis-induced AEC injury by inhibiting HMGB1-mediated activation of downstream inflammasomes and signaling pathways, thereby alleviating bacterial Sepsis-Associated ALI. Additionally, increased serum HMGB3 mRNA levels in bacterial Sepsis-Associated ALI patients, along with the involvement of the miR-424-5p/HMGB3 axis in sepsis-induced AEC inflammation and apoptosis (117), suggest that targeting HMGB proteins may offer a novel therapeutic approach for managing AEC dysfunction in sepsis. Targeting HMGB proteins or their signaling pathways may provide new therapeutic strategies for the treatment of bacterial Sepsis-Associated ALI. However, a deeper understanding of the molecular mechanisms by which HMGB proteins regulate inflammation and apoptosis, as well as the clinical validation of potential drugs and biomarkers, is essential for developing effective treatments. As research progresses, it may become possible to design novel therapies specifically targeting HMGB1, HMGB3, and their associated pathways to alleviate the impact of bacterial Sepsis-Associated ALI.

### 4.5 TLR

TLR, an evolutionarily conserved family within the innate immune system, act as the first line of defense against microbial pathogens by recognizing pathogen-associated molecular patterns (PAMPs). Dysregulated TLR responses contribute to a range of inflammatory and immune disorders (118). During bacterial Sepsis-Associated ALI, NETs regulate AEC functionality and disease pathogenesis through TLR9 pathway activation, which subsequently modulates METTL3-dependent methylation patterns (91). Furthermore, endotoxin exposure upregulates TLR2 expression (95), initiating a downstream signaling cascade through MyD88, mitogen-activated protein kinase 4 (MEK4), JNK1, and AP-1. This signaling axis stimulates alveolar epithelial cells (AECs) to overproduce surfactant protein-A (SP-A), thereby exacerbating the pathogenesis of bacterial Sepsis-Associated ALI (119). Together, these findings highlight the significant role of TLR activation in influencing AEC function and surfactant protein production during bacterial Sepsis-Associated ALI. These insights suggest that targeted modulation of TLR expression could provide a promising therapeutic approach for managing bacterial Sepsis-Associated ALI (87). Further research could focus on developing drugs that target TLRs to intervene and alleviate sepsis-associated AEC injury.

### 4.6 Methylation and METTL

The heterodimeric core complex consisting of methyltransferase-like 3 and 4 (METTL3/4) catalyzes N6-methyladenosine (m6A) methylation, a prevalent modification in mammalian messenger RNA (mRNA) and non-coding RNA. This modification plays a critical role in regulating mRNA stability, processing, and translation (120). In the context of bacterial sepsis, lactate and NETs upregulate METTL3 expression by activating p300 histone acetyltransferase-mediated enhancer modifications (H3K27ac and H3K18la) (3, 53). These changes promote m6A methylation of mRNAs such as Acyl-CoA Synthetase Long-Chain Family Member 4 (ACSL4), HIF-1α, GPX4, and SIRT1, stabilizing ACSL4 and HIF-1α transcripts while enhancing degradation of GPX4 and SIRT1 mRNAs. This molecular regulation contributes to AEC ferroptosis and autophagic dysfunction in bacterial Sepsis-Associated ALI (121). Moreover, METTL4 exacerbates endotoxin-induced AEC mitochondrial dysfunction and ferroptosis by enhancing the interaction between YTH N6-Methyladenosine RNA Binding Protein 2 (YTHDF2) and NRF2, leading to increased m6A modification and degradation of NRF2 mRNA (9). DNA methyltransferase 1 (DNMT1) further amplifies inflammatory responses through the DNMT1/miR-130a/Zinc Finger E-Box Binding Homeobox 1 (ZEB1) axis, promoting the secretion of inflammatory factors from AECs (30). In summary, methyltransferase-mediated modifications critically regulate mRNA metabolism and contribute to sepsis-induced AEC injury and inflammation. Targeted modulation of methyltransferase activity may offer a promising therapeutic strategy to alleviate AEC dysfunction and mitigate the progression of bacterial Sepsis-Associated ALI (120, 122). Targeting methylation-modifying enzymes, such as METTL3, METTL4, and DNMT1, provides a promising therapeutic strategy to regulate gene expression, alleviate AEC dysfunction, and slow the progression of bacterial Sepsis-Associated ALI. However, the precise molecular mechanisms by which these modifications regulate cellular responses in sepsis remain to be explored, making this an essential area for future research.

### 4.7 MAPK

MAPK family consists of a series of signaling molecules that transduce signals from the cell surface to the nucleus. MAPKs play a crucial role in various cellular processes, including proliferation, stress responses, inflammation, differentiation, transformation, apoptosis, and other signaling pathways. In mammals, the conventional MAPK family consists of four subfamilies: ERK, JNK, p38, and ERK5. The MAPK/ERK pathway is primarily involved in cell proliferation, differentiation, and the activation of various growth factor receptors. The MAPK/p38 pathway mainly mediates cellular responses related to inflammation and apoptosis, while the MAPK/JNK pathway participates in cellular stress responses such as radiation and osmotic stress (123).

In bacterial Sepsis-Associated ALI, the MAPK signaling pathway critically regulates NF-κB activation and phosphorylation, contributing to disease pathogenesis (for detailed mechanisms, refer to the section on NF-κB and Phosphorylation). Notably, studies have demonstrated that suppressing histone deacetylase 4 (HDAC4) expression attenuates LPS-induced alveolar epithelial cell (AEC) inflammation and oxidative stress by inhibiting the JNK/AP-1 signaling axis. This intervention improves mitochondrial function and reduces AEC apoptosis (124). Sea buckthorn has been shown to inhibit the LPS-induced upregulation of MAPK3 expression in AECs, suppress ferroptosis, and enhance cell vitality (54). Studies in sepsis patients with COVID-19 pneumonia have also found that Rapidly Accelerated Fibrosarcoma (RAF)/MEK/ERK pathway activation may be associated with immune system recovery, correlating with patient survival rates (125). In summary, the MAPK signaling pathway plays a significant role in regulating AEC inflammation and cell injury in bacterial Sepsis-Associated ALI. Modulating the MAPK signaling pathway offers a potential therapeutic approach to improve AEC damage in bacterial Sepsis-Associated ALI. However, the specific molecular mechanisms and potential drug candidates remain to be further explored through in-depth research.

### 4.8 Ion channels

Vascular leakage and alveolar edema are significant pathological changes in bacterial Sepsis-Associated ALI. In bacterial Sepsis-Associated ALI, endotoxins reduce the expression and increase the degradation of ion channels and water channel proteins in AECs, including ENaC, Na,K-ATPase AQP1, and AQP5, leading to decreased alveolar fluid clearance, increased pulmonary edema, and lung exudation, thus exacerbating bacterial Sepsis-Associated ALI (64, 65). Postmortem examinations of patients with diffuse alveolar damage have also shown an increased expression of AQP3, AQP5, and Na-K-ATPase, and a decreased expression of ENaC (126). Modulating the expression of water channel proteins, ENaC, and Na+,K+-ATPase can reduce pulmonary edema and lung tissue pathology by influencing alveolar fluid clearance, thus improving lung compliance, lung function, and survival rates in experimental animals (127, 128). Additionally, studies have found that water channel proteins such as AQP4 and AQPs are associated with inflammasome activation, while AQP3, AQP7, AQP9, and AQP10 enhance glycolysis during sepsis by promoting glycerol transport, supporting glucose uptake, and interacting with various metabolic signaling pathways, thereby regulating the immune cell energy supply and immune metabolism (129–131). In summary, ion channel-related proteins play a crucial role in regulating alveolar fluid balance, immune cell activation, and inflammatory responses in bacterial Sepsis-Associated ALI. However, the relationship and mechanisms by which these proteins modulate immune cell activation and inflammation, in connection to AEC damage in bacterial Sepsis-Associated ALI remain to be further explored.

### 4.9 Ubiquitination

Ubiquitination is a key post-translational modification regulated by the coordinated action of E1 ubiquitin-activating enzymes, E2 ubiquitin-conjugating enzymes, and E3 ubiquitin ligases. This process facilitates the covalent attachment of ubiquitin to target proteins, influencing signaling pathways and the assembly or degradation of protein complexes (132). In bacterial Sepsis-Associated ALI, several ubiquitination-related enzymes have been implicated in AEC injury and inflammation. These include ubiquitin-specific peptidases (USP10, USP38) (71, 133), E3 ubiquitin ligases, including S-phase kinase-associated protein 2 (SKP2) and F-box/WD repeat-containing protein 7 (FBXW7) (51, 103), and the trimeric E3 ligase family member Ring finger protein 99 (98). These enzymes regulate the ubiquitination and deubiquitination of key proteins such as GPX4, NLRP3, and AUF1, with their modulation offering potential therapeutic benefits in mitigating AEC injury and bacterial Sepsis-Associated ALI progression. Additionally, erythropoietin (EPO) has been shown to inhibit endotoxin-induced ubiquitin-mediated degradation of ENaC and Na,K-ATPase in AEC II via the EPOR/JAK2/STAT3/SGK1/Nedd4-2 pathway. This intervention enhances alveolar fluid clearance, alleviates bacterial Sepsis-Associated ALI pathology, restores pulmonary function, and improves survival outcomes in experimental models (63). Collectively, these findings highlight the therapeutic potential of targeting ubiquitination pathways in bacterial Sepsis-Associated ALI, with beneficial effects observed in protein homeostasis regulation, AEC protection, inflammation reduction, and alveolar fluid balance restoration. The ubiquitination regulatory system plays a dual role in the pathogenesis of bacterial Sepsis-Associated ALI: it contributes to the amplification of harmful inflammation and apoptosis, while also exerting protective effects by regulating protein stability and function to promote alveolar repair and fluid clearance. Therefore, precise modulation of ubiquitination enzymes and their associated signaling pathways may offer novel strategies for the intervention and treatment of ALI.

### 4.10 Acetylation

Acetylation and deacetylation exhibit target diversity and are post-translational modifications closely linked to metabolism. They are highly sensitive to the concentrations of acetyl-CoA, acyl-CoA, and Nicotinamide Adenine Dinucleotide (NAD), playing critical roles in various cellular and physiological processes such as transcription, autophagy, mitosis, and differentiation (134). These modifications are involved in the pathogenesis of bacterial sepsis-induced organ dysfunction, contributing to progression of the disease (135). In sepsis-mediated AEC injury, histone deacetylases Histone Deacetylase 3 (HDAC3) (60) and SIRT1 (69, 121) play key roles in regulating AEC autophagy, oxidative stress, mitochondrial dysfunction, and inflammatory factor release, thereby influencing AEC viability. Notably, HDAC3 also suppresses the expression of intercellular junction proteins, compromising epithelial integrity, increasing permeability, and exacerbating bacterial Sepsis-Associated ALI progression (60). Conversely, SIRT3 exerts protective effects by inhibiting lipopolysaccharide-induced acetylation of OPA1 at K792 in alveolar macrophages (77) and promoting the deacetylation of Superoxide Dismutase 2 (SOD2) at K122/K68 in AECs (136). These mechanisms contribute to mitochondrial quality control, suppress macrophage polarization, and regulate AEC autophagy and fatty acid oxidation. Ultimately, SIRT3 activity reduces oxidative stress, mitigates cellular damage, and preserves barrier function, thereby alleviating bacterial Sepsis-Associated ALI. These findings underscore the importance of acetylation and deacetylation dynamics in bacterial sepsis, which modulate autophagy, oxidative stress, and mitochondrial dysfunction to influence AEC inflammatory responses, injury, and barrier integrity. Targeting this intricate post-translational regulatory network represents a promising therapeutic strategy for bacterial Sepsis-Associated ALI management.

### 4.11 Exosomes

**Figure 3:**
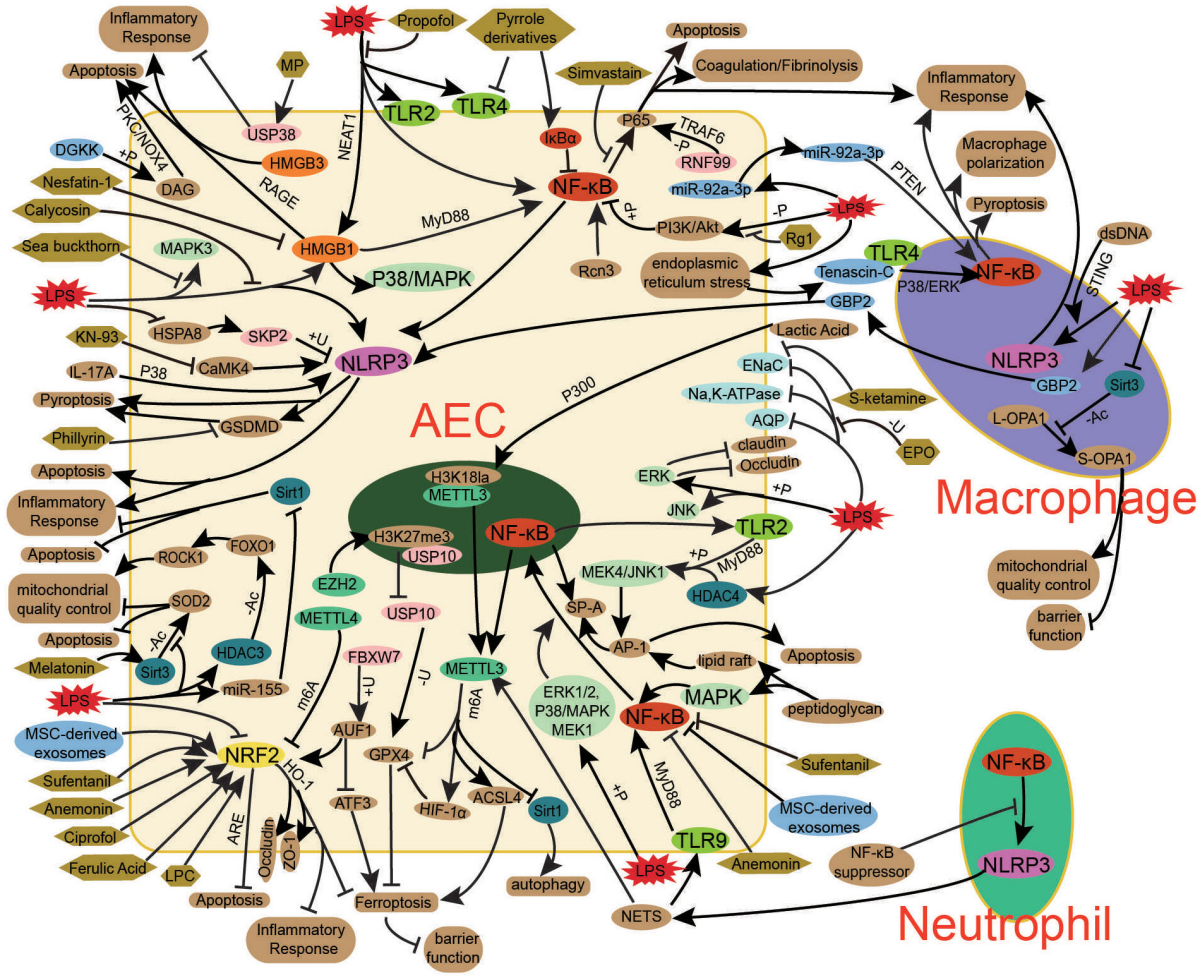
Key mechanisms in bacterial sepsis-associated ALI. The pathogenesis of bacterial sepsis-associated ALI involves a complex interplay of key signaling pathways (NF-κB, NRF2, NLRP3, HMGB, TLR, MAPK), post-translational modifications (phosphorylation, methylation, ubiquitination, acetylation), and exosome-mediated intercellular communication, which collectively contribute to AEC injury and dysfunction. NF-κB drives inflammatory responses through cytokine production and NLRP3 inflammasome activation, while NRF2 regulates antioxidant defense against oxidative stress and ferroptosis. HMGB1-RAGE signaling and TLR activation further amplify inflammation and cell death pathways. Post-translational modifications, including METTL3/4-mediated m6A methylation and SIRT/HDAC-dependent acetylation, fine-tune these processes by modulating mRNA stability and protein function. AEC-derived exosomes propagate injury through immune cell activation, whereas MSC exosomes exert protective effects. Therapeutic interventions targeting these mechanisms - including small molecule inhibitors (NF-κB, NLRP3), NRF2 activators, methylation regulators, ion channel modulators (ENaC/AQP), and biologics (LL-37, MSC exosomes) - offer promising approaches to mitigate AEC damage and restore alveolar function in sepsis-associated ALI. The integrated modulation of these interconnected pathways represents a viable strategy for developing effective treatments.

Exosomes, small extracellular vesicles involved in intercellular communication, mediate the transfer of proteins, lipids, and nucleic acids, influencing immune responses, metabolic reprogramming, coagulopathy, and organ dysfunction in bacterial sepsis (137). Studies indicate that serum exosomes from sepsis patients induce oxidative stress, inflammation, and AECs injury via the Diacylglycerol Kinase (DGK)/Diacylglycerol (DAG)/Protein Kinase C (PKC)/NADPH Oxidase 4 (NOX4) pathway (25). Additionally, endotoxin-stimulated alveolar macrophages release exosomes that activate AEC inflammasomes, promoting inflammatory responses (76). In turn, AEC-derived exosomes amplify pulmonary inflammation by activating inflammatory signaling in macrophages, exacerbating mitochondrial damage, and inducing macrophage apoptosis—processes that may be mitigated by inhibiting AEC exosome release, a potential therapeutic approach for bacterial Sepsis-Associated ALI (78, 79). Furthermore, MSC cell-derived exosomes have shown protective effects by maintaining mitochondrial homeostasis, suppress lipopolysaccharide-induced inflammatory cytokine release in AECs through NRF2 and NF-κB-related signaling pathways, thereby alleviating bacterial Sepsis-Associated AEC apoptosis and promoting cell proliferation (88, 108). Collectively, these findings highlight the role of exosomes in bacterial Sepsis-Associated ALI pathogenesis and suggest their potential as both biomarkers of bacterial sepsis-induced AEC damage and therapeutic agents. Modulating endogenous exosome secretion or administering exogenous exosomes may represent a promising strategy for bacterial Sepsis-Associated ALI treatment (**Figure 3**).

## 5 Treatment progress

### 5.1 Repair and functional regulation of AECs

As discussed, bacterial sepsis-induced AEC injury is a crucial mechanism in the development of bacterial Sepsis-Associated ALI. Research has identified several therapeutic agents that alleviate AEC apoptosis and ferroptosis, thus exerting protective effects on the lungs and mitigating the severity of bacterial Sepsis-Associated ALI. For example, metformin (138), bovine alveolar surfactant (47), ulinastatin (143), simvastatin (86), pyrrole derivatives (87), ginsenoside Rg1 (97, 144), recombinant Klotho protein (48) and Ciprofol (106) have all demonstrated lung-protective effects. Traditional Chinese medicines, including sea buckthorn (54) and shikonin (55), inhibit AEC ferroptosis, while the human antimicrobial peptide LL-37 (49) and natural lignan phillyrin (PHI) derived from *Forsythia suspensa* (50) suppress AEC pyroptosis, thus reducing AEC injury and mitigating inflammatory responses in bacterial Sepsis-Associated ALI. Antioxidants such as N-acetylcysteine (NAC) (35), melatonin (136), and tea polyphenols (139) have shown efficacy in attenuating bacterial Sepsis-Associated ALI by reducing ROS and RNS production, thus alleviating oxidative stress and improving mitochondrial function. Additionally, the omega-3 fatty acid-derived lipid mediator resolvin E1 (RvE1) has demonstrated protective effects against inflammation-induced mitochondrial dysfunction in AECs during severe inflammatory states (140). Furthermore, small-molecule modulators targeting specific signaling pathways, including the α7 Nicotinic Acetylcholine Receptor (α7nAChR) agonist PNU-282987 (141), miR-155 inhibitor (69), Receptor-Interacting Protein Kinase 3 (RIPK3) inhibitor UH15-38 (15), Glycogen Synthase Kinase-3 Beta (GSK-3β) inhibitor Tideglusib (138), and glycolysis inhibitor 3PO (42), have shown promise in improving bacterial Sepsis-Associated ALI outcomes through the regulation of AEC activity. In summary, therapeutic strategies aimed at modulating AEC activity, oxidative stress responses, and mitochondrial function offer promising approaches for mitigating AEC injury and promoting cellular repair and regeneration in bacterial Sepsis-Associated ALI. Clinical studies have also shown that the combination of ginsenoside Rg1 and ulinastatin in treating sepsis-associated ALI can significantly improve hemodynamic and pulmonary circulation parameters, such as cardiac index, intrathoracic blood volume index, and central venous pressure (142). However, the lack of clinical trial validation presents significant translational challenges, including the need for optimal drug selection and rigorous preclinical evaluation to ensure clinical safety and efficacy. This gap underscores the need for extensive future research to bridge the divide between preclinical findings and clinical application. In summary, therapeutic strategies aimed at modulating AEC activity, oxidative stress, and mitochondrial function to alleviate AEC injury and promote cellular repair and regeneration represent a promising approach to treating bacterial Sepsis-Associated ALI. However, most of these drugs currently lack relevant clinical randomized controlled trials. The selection of optimal drugs, drug formulations, and treatment plans, as well as the evaluation of their clinical safety and efficacy, still require further research. There is still a long way to go before these approaches can be applied in clinical settings.

Impairment of AEC barrier function is also a significant cause of pulmonary inflammatory exudation in bacterial sepsis. In bacterial Sepsis-Associated ALI, inhibiting the degradation of tight junction proteins or using ferulic acid (FA) to improve the expression of tight junction proteins in bacterial sepsis-associated AECs can improve AEC barrier dysfunction and alleviate lung tissue damage (104, 109). Similarly, antimicrobial therapies and microbiota modulation can regulate junctional proteins, improving bacterial Sepsis-Associated ALI and increasing survival rates in experimental models (59). Additionally, pharmacological agents such as dexamethasone (62), erythropoietin (EPO) (63), and the anti-inflammatory formulation Yan Tiao Decoction (65) have been reported to regulate ion channels in AECs, including ENaC, Na,K-ATPase, NKAα1, NKAβ1, AQP1, and AQP5. These agents promote alveolar fluid clearance, thereby mitigating the pathological effects of bacterial Sepsis-Associated ALI. These findings suggest that modulating the expression of both AEC junctional proteins and ion channels through pharmacological interventions to restore alveolar barrier function and promote fluid resolution may be one of the potential therapeutic strategies for bacterial Sepsis-Associated ALI. However, key issues such as drug selection and intervention timing still require further in-depth research (**Figure 3**).

### 5.2 AEC-related inflammation regulation

Bacterial sepsis-induced dysregulation of inflammatory cytokine release from AECs and their interactions with immune cells play a critical role in the pathogenesis of pulmonary inflammation. Recent studies have highlighted the potential of anti-inflammatory agents, including methylprednisolone (71, 145), dexamethasone (62), propofol (89), sufentanil (105), calycosin (72), Klotho (48), Ciprofol (106), and Anemonin (93) to attenuate septic lung inflammation and injury. These agents act by suppressing inflammasome activation and downstream signaling pathways in AECs, thereby reducing pro-inflammatory cytokine production. Additionally, MSC-derived exosomes have been shown to inhibit LPS-induced inflammatory cytokine release from AEC II and promote cellular proliferation (88). Small-molecule modulators targeting specific signaling pathways—such as the PKC inhibitor LXS-196 (Darovasertib), novel NOX4 inhibitor GLX351322 (25), Calcium/Calmodulin-Dependent Protein Kinase IV (CaMK4) inhibitor KN-93 (14), α7nAChR agonist PNU-282987 (141), NF-κB suppressor Bay-117082 (90), glycolysis inhibitor 3PO (42), and miR-155 inhibitor (69)—can also mitigate septic pulmonary inflammation and lung injury by modulating AEC inflammatory signaling and cytokine release. Further research on exosomes has revealed their involvement in septic pulmonary inflammation. Inhibition of AEC-derived exosome secretion reduces macrophage-mediated lung inflammation (82), while MSC-derived exosomes regulate AEC inflammatory factor release in bacterial Sepsis-Associated ALI (88). However, the full mechanistic details of these processes remain to be elucidated. In addition, inflammatory damage to AECs caused by Gram-positive bacteria is also one of the important causes of bacterial Sepsis-Associated ALI, but related therapeutic agents have not been extensively studied.

Collectively, these findings indicate that pharmacological agents, small-molecule pathway modulators, and exosome-based strategies targeting AEC-immune cell interactions and inflammatory mediator release have the potential to effectively ameliorate bacterial sepsis-induced pulmonary inflammation and the severity of bacterial Sepsis-Associated ALI. Some clinical studies have shown that the anti-inflammatory drug hydrocortisone can reduce the 28-day mortality rate in patients with severe community-acquired pneumonia (146). However, studies on bacterial sepsis-associated ARDS have not yielded significant positive results (147–149), which may be attributed to the heterogeneity between different etiologies of bacterial sepsis-associated ARDS, as well as inconsistencies in drug regimens and intervention timing. Further research is needed to explore the optimal therapeutic approaches and timing for intervention. Additionally, a retrospective analysis of the effects of drugs currently used in clinical practice, such as methylprednisolone, dexamethasone, propofol, and sufentanil, on the inflammatory response in bacterial Sepsis-Associated ALI patients could help promote further clinical research and application of these medications (**Table 1**).

**Table 1:** Therapeutic strategies and treatment progress for AEC injury in bacterial sepsis-associated ALI.

Drug	Types	Model	Mechanism of action and targets	Reference
Metformin	Compound	P. aeruginosa-induced pneumonia in mice	Cell Repair and Regeneration (AMPK)	Becker E 2023 (138)
pulmonary surfactant	Biological Products	LPS-induced rat	Cell Repair and Regeneration	Chen X 2024 (47)
Ulinastatin	Compound	CLP-induced rat	Cell Repair and Regeneration	Li Y 2018 (143)
Simvastatin	Compound	LPS-induced rat	Cell Repair and Regeneration(Survivin/NF-κB/p65)	Nežić L 2022 (86)
Pyrrol derivates	Compound	LPS-induced A549	Cell Repair and Regeneration	Cabrera-Benítez N E 2016 (87)
Ginsenoside Rg1	TCM Extracts	CLP-induced mouse, LPS-induced MLE 12	Cell Repair and Regeneration (PI3K-Akt and PERK/eIF2α/ATF4/CHOP)	Zhong K 2024 (97), Zhong K 2024 (144)
Sea buckthorn	TCM	CLP-induced mice	Cell Repair and Regeneration (*IL1B*, *MAPK*3, *TXN*	Li M 2025 (54)
Shikonin	TCM	LPS-induced mice, LPS-induced mice AECII	Cell Repair and Regeneration (TMEM16A)	Jiang W 2023 (55)
Phillyrin	TCM Extracts	CLP-induced mice, LPS-induced MLE 12	Cell Repair and Regeneration (NLRP3/caspase-1/GSDMD)	Ji C 2024 (50)
N- acetylcysteine	Compound	LPS-induced A549	Cell Repair and Regeneration (Cytochrome c, Caspase)	Chuang C Y 2011 (35)
Melatonin	Compound	CLP-induced mice, LPS-induced A549	Cell Repair and Regeneration (SIRT3, SOD2)	Ning L 2022 (136)
Tea Polyphenols	Natural Extracts	CLP-induced rat, LPS-induced L2	Cell Repair and Regeneration (DJ-1)	Jia C M 2021 (139)
Resolvin E1	Compound	TNF α-induced A549	Cell Repair and Regeneration	Mayer K 2019 (140)
CRAMP (LL-37)	Biological Products	LPS-induced mice, LPS-induced A549	Cell Repair and Regeneration(NLRP3, GSDMD)	Wang Q 2024 (49)
Recombinant Klotho protein	Compound	CLP-induced mouse, LPS-induced HPAEpiCs	Cell Repair and Regeneration, Modulation of Inflammatory Response (Bcl-2/Bax/caspase-3)	Li X B 2024 (48)
Ciprofol	Compound	LPS-induced mice, LPS-induced MLE12	Cell Repair and Regeneration, Modulation of Inflammatory Response (Nrf2)	Zhao Q 2024 (106)
Methylprednisolone	Compound	LPS-induced mice, LPS-induced mice AECII	Modulation of Inflammatory Response (miR-151-5p/USP38, *SNHG5/CPNE1*)	Yuan Z 2024 (71), Zhang L 2021 (145)
Propofol	Compound	LPS-induced rat, LPS-induced HPAEpiCs	Modulation of Inflammatory Response (HMGB1, TLR2/4, NF-κB)	Wang X 2016 (89)
Sufentanil	Compound	CLP-induced rat, LPS-induced A549	Modulation of Inflammatory Response (KNG1, NF-κB, Nrf2/HO-1)	Hu Q 2020 (105)
Calycosin	TCM Extracts	CLP-induced rat, LPS-induced rat AECII	Modulation of Inflammatory Response (HMGB1/MyD88/NF-κB, NLRP3)	Chen G 2021 (72)
Msc exosomes	Biological Products	LPS-induced MLE12	Modulation of Inflammatory Response	Li J 2020 (88)
Anemonin	Compound	LPS-induced mice, LPS-induced MLE12	Modulation of Inflammatory Response (NF-κB, Nrf2/HO-1)	Xia Q 2025 (93)
Dexamethasone	Compound	LPS-induced AEC-II of newborn piglets	Modulation of Inflammatory Response, Barrier Function Repair and Enhancement	He L 2018 (62)
Ferulic acid	Compound	CLP-induced mice, LPS-induced MLE12	Barrier Function Repair and Enhancement (Nrf2/HO-1)	Tang X 2022 (104)
EPO	Biological Products	LPS-induced rat, LPS-induced rat AECII	Barrier Function Repair and Enhancement (ENaC, Na,K-ATPase)	Gao Y 2024 (63)
Yantiao Decoction	TCM	CLP-induced rat LPS-induced RLE-6TN	Barrier Function Repair and Enhancement (α-ENaC, NKAα1, NKAβ1,AQP1, AQP5, SP-D)	Xu M 2023 (65)

LPS, lipopolysaccharide; CLP, Cecal ligation and puncture; TCM, Traditional Chinese Medicine; MSC, Mesenchymal stem cell; EPO, Erythropoietin.

## 6 Conclusion and future perspectives

In this review, we have systematically summarized the pathophysiological alterations of AECs in bacterial Sepsis-Associated ALI, highlighted key molecular mechanisms, and evaluated current therapeutic advancements. This work provides a foundation for further exploration of bacterial sepsis-induced AEC dysfunction and development of targeted interventions aimed at protecting AECs to mitigate bacterial ALI progression.

AECs, as essential components of the alveoli, not only maintain normal alveolar function but also actively participate in inflammatory responses during injury and subsequent repair processes. Bacterial sepsis damages AECs through mechanisms such as oxidative stress, cell death, and barrier dysfunction, while bacterial sepsis-activated AECs exacerbate bacterial Sepsis-Associated ALI by releasing inflammatory mediators and interacting with immune cells. Although current research involves a variety of substances, including traditional drugs, Chinese herbal extracts, novel compounds, small-molecule modulators, and exosomes, certain gaps still remain.

First, the drugs currently being researched, such as Chinese herbal extracts and novel compounds, lack standardized, large-scale preparation methods and unified drug regimens. This results in potential discrepancies between research findings. Furthermore, most of these drugs have not undergone pharmacokinetic studies, indicating a significant gap before further clinical trials and mechanistic exploration can take place.

Second, due to the unclear mechanisms and therapeutic efficacy of certain drugs, most studies are limited to basic research. The few existing clinical studies mainly focus on new uses for previously used drugs, and no consistent results have been achieved. Thus, more in-depth investigation into the specific mechanisms of these drugs is required. Additionally, retrospective analyses of the impact of widely used clinical drugs, such as metformin, ulinastatin, simvastatin, sufentanil, propofol, and Ciprofol, on the prognosis of ARDS patients could promote further research and clinical application of these therapies. However, the clinical translation of new drugs discovered through basic research requires long-term exploration and validation.

Moreover, sepsis is a heterogeneous syndrome involving complex pathophysiological processes (150). Most current studies use LPS and CLP models, which may introduce discrepancies due to variations in the extent of injury caused by various operators. Furthermore, these models only simulate bacterial Sepsis-Associated ALI caused by endotoxins from Gram-negative bacteria. More relevant models need to be developed to study ALI induced by other Gram-negative bacteria, Gram-positive bacteria, and bacterial exotoxins.

Finally, the effective prevention and treatment of sepsis-related organ dysfunction require early recognition and intervention. Developing reliable methods to detect or predict sepsis-induced AEC injury, thereby preventing and treating SARDS and improving patient prognosis, remains an area that requires further research (151). Investigating its underlying mechanisms and developing new biomarkers for early detection would also be crucial.

In conclusion, AECs are closely related to bacterial Sepsis-Associated ALI. Promoting their activity and functional recovery, along with regulating related inflammatory responses, is one of the effective strategies for treating this condition. However, further research into the specific mechanisms and drugs targeting AECs is necessary for effective clinical application.
